# Competing in Hot Conditions at the Tokyo Olympic Games: Preparation Strategies Used by Australian Race Walkers

**DOI:** 10.3389/fphys.2022.836858

**Published:** 2022-03-23

**Authors:** Amelia J. Carr, Brent S. Vallance, Jessica Rothwell, Anna E. Rea, Louise M. Burke, Joshua H. Guy

**Affiliations:** ^1^Centre for Sport Research, School of Exercise and Nutrition Sciences, Deakin University, Melbourne, VIC, Australia; ^2^Athletics Australia, Melbourne, VIC, Australia; ^3^Exercise and Nutrition Research Program, Mary Mackillop Institute for Health Research, Australian Catholic University, Melbourne, VIC, Australia; ^4^Victorian Institute of Sport, Melbourne, VIC, Australia; ^5^School of Health, Medical and Applied Sciences, Central Queensland University, Cairns, QLD, Australia

**Keywords:** Tokyo 2021, heat acclimation, heat acclimatization, passive heat exposure, hyperhydration, periodized nutrition

## Abstract

**Introduction:**

The Tokyo 2021 Olympic Games was anticipated to expose athletes to the most challenging climatic conditions experienced in the history of the modern Olympic Games. This study documents strategies executed by Australian endurance athletes during the team holding camp and Olympic Games experiences, including (1) baseline physiological data, training data, and heat acclimation/acclimatization practices; (2) pre- and in-race cooling and nutritional strategies, and (3) Olympic Games race performance data.

**Methods:**

Six athletes (three males, three females; age 24 ± 4 years; VO_2max_ 63.2 ± 8.7 mL⋅kg^–1^⋅min^–1^; sum of 7 skinfolds 53.1 ± 23.4 mm) were observed prior to and during the team holding camp held in Cairns, QLD, Australia. Athletes completed 6–7 weeks of intermittent heat acclimation training, utilizing a combination of 2–4 passive and active acclimation sessions per week. Active acclimation was systematically increased via exposure time, exercise intensity, temperature, and humidity. In the team holding camp, athletes undertook a further 23 heat acclimatization training sessions over 18 days in a continuous fashion. Hyperhydration (using sodium and glycerol osmolytes), and internal and external pre-and in-race cooling methods were also utilized. A low energy availability intervention was implemented with two athletes, as a strategy to periodize ideal race body composition. Race performance data and environmental conditions from the 2021 Olympic Games were also documented.

**Results:**

The highest values for aerobic capacity were 63.6 mL⋅kg^–1^⋅min^–1^ for female race walkers and 73.7 mL⋅kg^–1^⋅min^–1^ for males. Training volume for the six athletes was the highest in the second week of the team holding camp, and training intensity was lowest in the first week of the team holding camp. Performance outcomes included 6th place in the women’s 20 km event (1:30:39), which was within 2% of her 20 km personal best time, and 8th place in the men’s 50 km event (3:52:01), which was a personal best performance time.

**Conclusion:**

Periodized training, heat acclimation/acclimatization, cooling and nutritional strategies study may have contributed to the race outcomes in Olympic Games held hot, humid conditions, for the race walkers within this observational study.

## Introduction

It was projected that the Tokyo Olympic Games would expose athletes to the most challenging climatic conditions ever observed in the history of the modern Olympic Games (Szubski, 2016; Racinais and Ihsan, 2020). In response, the International Olympic Committee adopted countermeasures for race walking and marathon events (International Olympic Committee, 2019). Race venues were changed from Tokyo to Sapporo, given that ambient temperature in Sapporo is typically lower than in Tokyo. Further, even after the events were relocated, the risk of heat illness remained due to the potential for athletes’ exposure to substantial thermal load in Sapporo. Therefore, in Sapporo, altered event start times were implemented, to reduce risks of heat-related illness, based upon historical weather data (Takayama et al., 2020; Seo and Honjo, 2021). It has been well established that the performance of elite endurance athletes is compromised in hot environments and it is therefore important for athletes to prepare for endurance events in environmentally challenging conditions (Guy et al., 2015). The planning and preparation undertaken by elite athletes for events in hot and/or humid conditions typically include periodized environmental training practices such as heat acclimation (repeated heat exposures within a heat chamber or heated room) and acclimatization (living and training in a natural similar environment to the competition), and in some cases passive heat exposure (passive heat acclimation), achieved via sauna bathing or hot water immersion in either an intermittent or continuous approach (Périard et al., 2015; Mujika et al., 2019; Racinais et al., 2019; Saunders et al., 2019). Strategies used by athletes to lessen the expected increases in core body temperature and skin temperature when competing in hot, humid conditions may also include internal cooling methods including ice slurry ingestion and menthol gel ingestion, and external cooling methods such as cold water immersion and application of ice and cold water to the skin (Ross et al., 2013; Stevens and Best, 2017; Burke et al., 2019b; Périard et al., 2021). Recently, the use of such strategies by elite race walkers and marathon runners has been documented during international championship events (Stevens et al., 2020; Racinais et al., 2021).

Prior to major international championship events, national sporting organizations typically coordinate team holding camps in similar environmental conditions to those expected for the championship (Maughan, 1997; Arnold et al., 2015). Preparation camps for endurance athletes often comprise a combination of key high-intensity sessions, and lower intensity and longer duration endurance sessions, and present opportunities to profile athletes’ physiological parameters immediately prior to important international championships (Pugliese et al., 2014; Tønnessen et al., 2014; Carr et al., 2020; Racinais et al., 2021). Additionally, preparation camps provide opportunities to adapt and refine intervention strategies under the guidance of appointed team staff (Maughan, 1997; Arnold et al., 2015; Mujika et al., 2019). The postponed 2021 Tokyo Olympic Games incurred further changes to the planned dates and locations of camps due to the COVID-19 pandemic as a result of travel bans, restricted access to training venues and requirements for physical distancing (Fabre et al., 2020; Mulcahey et al., 2021; Shimizu et al., 2021). Australian athletes competing in race walking and marathon events faced the challenge of preparing in the cool conditions in the southern states of Australia in July and August 2021 for the hot, humid conditions forecast in Sapporo (Takayama et al., 2020; Carr et al., 2020; Wu et al., 2020; Seo and Honjo, 2021). Further, restricted access to the Sapporo Olympic Village due to COVID-19 meant that in some cases athletes could access their event location only several days prior to their events, in contrast to the several weeks’ training typically performed near race venues in a regular Olympic schedule (Carr et al., 2020; International Olympic Committee, 2021). The specific strategies adopted during preparation camps in response to the unique challenges of the Tokyo Olympic Games have yet to be comprehensively documented.

Nutritional strategies that improve hydration status and modify body composition can support athletes’ performance in hot, humid conditions (Burke et al., 2019b; Saunders et al., 2019; Areta et al., 2021). The addition of specific osmotic agents (e.g., glycerol and sodium) to a pre-event fluid bolus has been demonstrated to enhance hydration status and significantly increase pre-exercise blood plasma volume, significantly decrease core temperature during exercise, and modify body mass, urine specific gravity and plasma osmolality (Sims et al., 2007a,b; Goulet et al., 2018; Maughan et al., 2018; Saunders et al., 2019). Short-term exposure to low energy availability (LEA) can facilitate a reduction in body mass, which when periodized appropriately and managed by sports dietitians, might lead to improved performance in endurance events without the negative health implications of chronic LEA such as impaired endocrine and immune function, and impaired bone health (Stellingwerff, 2018; Burke et al., 2019b; Mujika et al., 2019; Areta et al., 2021). Nutritional and hydration strategies used by national teams within holding camps prior to Olympic Games have been previously reported (Maughan, 1997) as have outcomes of periodized nutritional strategies for individual athletes (Stellingwerff, 2018). However, there is limited documentation of the use of nutritional strategies by individual athletes during their final preparations for Olympic Games.

The aim of this study was to provide a comprehensive account of the preparation of elite endurance athletes during team holding camps, prior to international championship events held in hot, humid conditions. It provides specific documentation of the strategies undertaken by elite Australian male and female race walkers for the Tokyo 2021 Olympic Games: (1) baseline physiological data, training data and heat acclimation/acclimatization practices; (2) cooling strategies and nutritional strategies, and (3) Olympic Games race performance.

## Materials and Methods

### Design

**FIGURE 1:**
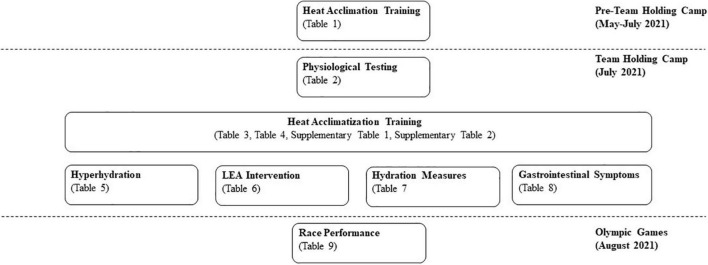
Schematic of activities for Australian race walkers (*n* = 6) prior to and during the team holding camp, and during the Tokyo Olympic Games. LEA; low energy availability.

An observational study was conducted during the holding camp for the Australian athletics team (Cairns, QLD, Australia, July 2021) prior to the Tokyo 2021 Olympic Games. Physiological data, training data, details of heat acclimation/acclimatization practices, cooling and nutritional intervention strategies and Olympic Games performance outcomes were documented (Figure 1). The team holding camp was held in Cairns, QLD, Australia (12th–30th July 2021). Training sessions were conducted between 8:00 am and 10:00 am for morning sessions and between 4:00 pm and 6:00 pm for afternoon sessions. As such, the mean temperature and humidity values (12th–30th July 2021) were 23.0 ± 2.0°C and 71.4 ± 11.2% RH for the morning sessions and 24.3 ± 1.6°C and 67.4 ± 10.1% RH for the afternoon sessions. The mean of the maximal daily temperature and humidity values recorded for the period of 12th–30th July 2021 was 27.3 ± 1.1°C and 89.4 ± 9.4% RH (Australian Bureau of Meterology, 2021) which was similar to the mean maximal daily temperature and humidity values for Sapporo, Japan, for August, 2010–2020 (27.1 ± 1.2°C; 74.7 ± 3.0% RH; Japan Meteorological Agency, 2021), and therefore the venues for the 2021 Olympic Games race walking events.

### Participants

Data were collected for six elite-level athletes (three male and three female race walkers; age 24 ± 4 years; Table 1). All participants were Australian Olympic athletics team representatives. Experimental procedures were approved by the Central Queensland University Ethics Committee (approval no. 0000023260). Prior to the Olympic Games, the range of 20 km personal best performances (h) for females was 1:28:50–1:31:53; 1158-1104 World Athletics points, and the range of 20 km personal best performances for males was 1:20:19–1:24:27; 1184 - 1096 World Athletics points. The personal best performance for the only athlete who routinely competed in 50 km events (for males) was 3:52:58; 1145 World Athletics points (World Athletics, 2021).

**TABLE 1:** Example of training sessions performed for the 7 weeks prior to the team holding camp (*n* = 1; female).

	Monday	Tuesday	Wednesday	Thursday	Friday	Saturday	Sunday
**WEEK-11 Pre-Olympic Games; Volume = 134 km; 14.6% >4 mmol**⋅**L^–1^**							
**AM**	Easy Run + Gym	Chamber: 60 min* *10.0–10.3 km*⋅*h^–1^*	20 km walk	Easy Run + Gym	Repetitions: *1* × *2 km, 5* × *1 km*	Chamber: 60 min* *10.0–10.7 km*⋅*h^–1^*	30 km walk *4:58 min*⋅*km^–1^*
**PM**		Repetitions: *15* × *300 m*	Easy Run + Sauna*		Easy Run + Sauna*		Easy Run
**WEEK-10 Pre-Olympic Games; Volume = 125 km; 16.8% >4 mmol**⋅**L^–1^**							
**AM**	Easy Run + Gym	Chamber: 60 min* *10.0–11.0 km*⋅*h^–1^*	20 km walk *Fartlek*	Easy Run + Gym	Hill session *3 km, 2* × *2 km, 3 km, 6* × *500 m*	Chamber: 60 min* 10.0–11.0 km⋅h^–1^	25 km walk 4:52 min⋅km^–1^
**PM**		Easy Walk	Easy Run + Sauna*		Easy Run + Sauna*		Easy run
**WEEK-9 Pre-Olympic Games; Volume = 136 km; 14.6% >4 mmol**⋅**L^–1^**							
**AM**	Gym	Chamber: 60 min* *10.0–11.0 km*⋅*h^–1^*	25 km walk *5:00 min⋅km^–1^*	Easy Run + Gym	2 km + 6 × 500 m *4:15 min*⋅*km^–1^*	Chamber: 60 min* *10 km*⋅*h^–1^*	30 km walk *4:53 min*⋅*km^–1^*
**PM**		Repetitions: *12* × *400 m*	10 km + Sauna* *10 km*⋅*h^–1^*		Easy Run + Sauna*		Easy run
**WEEK-8 Pre-Olympic Games; Volume = 125 km; 18.4% >4 mmol**⋅**L^–1^**							
**AM**	Gym	Chamber: 60 min* *10.0–11.0 km*⋅*h^–1^*	Fartlek *20 km*	Easy Run + Gym	Repetitions: *6* × *2 km*	Chamber: 75 min* *13.4 km*⋅*h^–1^*	25 km walk *4:50 min*⋅*km^–1^*
**PM**		Easy walk	10 km + Sauna* *10 km*⋅*h^–1^*		Easy Run + Sauna*		Easy run
**WEEK-7 Pre-Olympic Games; Volume = 98 km; 29.6% >4 mmol**⋅**L^–1^**							
**AM**	Easy Run + Gym	Chamber: 20 min + 7 × 1 km* *(4:38 min*⋅*km^–1^)*	12 km walk 5:14 min⋅km^–1^	Easy Run + Gym	Hill session: *14* × *500 m*	Chamber: 75 min* *10.7 km*⋅*h^–1^*	15 km walk *4:37 min*⋅*km^–1^*
**PM**		Rest	6 km Walk *5:42 min*⋅*km^–1^*		6 km + Sauna* *10.0 km*⋅*h^–1^*		Easy Run
**WEEK-6 Pre-Olympic Games; Volume = 133 km; 12.0% >4 mmol**⋅**L^–1^**							
**AM**	Rest day	10 km walk	20 km walk *5:01 min*⋅*km^–1^*	30 min Run + Gym	Repetitions: *8* × *2 km*	Chamber: 75 min* *10.2 km*⋅*h^–1^*	30 km walk *5:04 min*⋅*km^–1^*
**PM**		Chamber: 60 min* *10.0–11.0 km*⋅*h^–1^*	Easy Run + Sauna*		Easy Run + Sauna*		Easy run
**WEEK-5 Pre-Olympic Games; Volume = 128 km; 15.2% >4 mmol**⋅**L^–1^**							
**AM**	Easy Run + Gym	Chamber: 60 min* *10.5–13.4 km*⋅*h^–1^*	25 km walk 5:00 min⋅km^–1^	Easy Run + Gym	Repetitions: *1 km* + *5* × *500 m*	Chamber: 75 min* *10.0–10.7 km*⋅*h^–1^*	25 km walk *4:54 min*⋅*km^–1^*
**PM**		Easy Walk	Easy Run + Sauna*		Easy Run + Sauna*		

*For each training week, volume was expressed as distance (km), and intensity was expressed as the percentage of training volume (km) >4 mmol⋅L^–1^ blood lactate concentration.*

*Sauna sessions commenced immediately post-training. Fluid was consumed ad libitum during sauna and heat chamber sessions.*

*Fluid intake was not quantified during sauna or heat chamber sessions.

### Methodology

#### Heat Acclimation Training

Prior to the team holding camp, participants’ weekly training programs were managed by Australian team and individual coaches. Typically, participants performed 1–2 race walking-specific heat acclimation sessions on a treadmill per week (intermittent HA), within their home training environment. For one of the female athletes, the duration (min), temperature (°C), relative humidity (% RH) and intensity (km⋅h^–1^) of active heat acclimation sessions were gradually increased across the 7 weeks prior to the holding camp from 60 to 75 min, 32–36°C and 60–70% RH, and treadmill speed was gradually increased from 10 to 13.4 km⋅h^–1^. Training session pace was recorded (h) for long duration sessions and key sessions, and distances were recorded for high intensity and low intensity sessions. For each training week, intensity was expressed as the percentage of training volume (km) >4 mmol⋅L^–1^ blood lactate concentration (Table 1).

#### Physiological Testing

Baseline physiological data were collected on the first day of the team holding camp to characterize participants prior to their departure for the Olympic Games. Anthropometric measures were recorded for each participant, including height using a portable stadiometer (Seca 213, Seca Asia Pacific, Kuala Lumpur, Malaysia), mass using body weight scales (A&D UC-321, Adelaide, SA, Australia) and sum of 7 skinfolds using skinfold calipers (Baty International, West Sussex, United Kingdom). An incremental, racewalking-specific treadmill test (Quinton TM55, Bothell, WA, United States) was performed, and submaximal economy data, quantified at 11 km⋅h^–1^ for females and 13 km⋅h^–1^ for males, was quantified according to a previously documented protocol (Saunders and Green, 2012). Blood lactate concentration (Lactate Pro, Arkray, Kyoto, Japan, heart rate (beats⋅min^–1^; Polar Electro Oy, Kempele, Finland) and oxygen consumption (Parvo Medics TrueOne 2400, Salt Lake City, UT, United States) were measured (Table 2).

**TABLE 2:** Baseline physiological data for athletes during the team holding camp (*n* = 6).

Parameter	Male (*n* = 3)	Female (*n* = 3)	Total (*n* = 6)
Age (y)	26 ± 5	22 ± 2	24 ± 4
Body mass (kg)	64.6 ± 1.6	53.1 ± 3.7	58.9 ± 6.8
Sum 7 skinfolds (mm)	38.4 ± 6.4	67.7 ± 26.2	53.1 ± 23.4
VO_2max_ (mL⋅kg^–1^⋅min^–1^)	70.0 ± 3.2	56.3 ± 6.3	63.2 ± 8.7
vVO_2max_ (km⋅h^–1^)	16.6 ± 0.9	15.1 ± 0.5	15.9 ± 1.1
v4 mmol⋅L^–1^ (km⋅h^–1^)	15.3 ± 0.6	13.5 ± 0.9	14.4 ± 1.2
4 mmol⋅L^–1^ (%VO_2max_)	89.0 ± 2.8	85.6 ± 4.8	87.3 ± 4.0
Submaximal economy (%VO_2max_)*	70.3 ± 7.4	66.0 ± 4.8	68.3 ± 5.9

*Submaximal economy data was quantified at 11 km⋅h^–1^ for females and 13 km⋅h^–1^ for males.

#### Heat Acclimatization Training

During the team holding camp, the typical weekly training involved six racewalking sessions, one running session, two resistance training sessions and cross-training sessions as required. All athletes (*n* = 6) completed 18 days’ heat acclimatization training during the team holding camp (Table 3) and was considered a continuous HA approach. At the time of the two key training sessions in the holding camp (16:00 h–18:00 h on 16th and 23rd July), the mean (±SD) temperature and humidity recorded across the two sessions was 24.6 ± 1.5°C; 73.5 ± 6.6% RH. These training sessions were conducted between 16:00 h–18:00 h to align with the race times of the 20 km events in the Olympic Games). The mean of the maximal temperature and humidity values on the dates of the two key training sessions was 27.6 ± 2.1°C; 97.5 ± 0.71% RH. Pre- and post-training body mass (kg) was recorded during these sessions. One of the female athletes completed an additional two passive heat acclimation sessions per week (Supplementary Table 1). While preparation for the 50 km race walking event involved long walking sessions of up to 33 km (Supplementary Table 2), other athletes undertook long walk sessions of 20–24 km and speed sessions involving 6–8 × 1–1.6 km repetitions (Table 4).

**TABLE 3:** Weekly training plan overview for race walking athletes (*n* = 6; three males, three females) during the team holding camp.

	Monday	Tuesday	Wednesday	Thursday	Friday	Saturday	Sunday
**WEEK-4 Pre-Olympic Games; Volume = ∼90 km**							
**AM**	Travel to Cairns	Easy 10 km walk	Lab testing	6 km Walk + Gym	Easy 12 km walk	Easy 12 km walk	20–32 km walk
**PM**	Gym	Training optional	Easy 8–12 km walk		Repetitions: *8* × 1,609 m*^†^		Repetitions: *10* × *300 m*
**WEEK-3 Pre-Olympic Games; Volume = ∼120 km**							
**AM**	Walk optional	12 km walk	22 km walk	Gym	8 km walk	Easy 12 km walk	25–30 km walk
**PM**	Gym	Repetitions: *16* × *400 m*	Easy 8 km (Walk/Run)		Repetitions: *10* × 1,200 m*^†^		Repetitions: *10* × *400 m*
**WEEK-2 Pre-Olympic Games; Volume = ∼72 km**							
**AM**	Walk optional	Warm-up	Easy 12–20 km walk	Gym	Warm-up	Travel to Sapporo	Warm-up
**PM**	Gym	Repetitions: *10* × *1 km*	Easy 8 km (Walk/Run)		Travel to Sydney	Light Training	Easy 6–8 km walk

*Hyperhydration trial. ^†^Pre-cooling trial.

**TABLE 4:** Training volume (h) and intensity (min⋅km^–1^) of key training sessions (6–8 × 1.2–1.6 km repetitions); 16th July and 23rd July 2021) during the team holding camp.

Athlete	Total repetitions duration (h)	Training pace (min⋅km^–1^)	Total session duration (h)	Work: Rest ratio
1	0:46:34.0 ± 10:23.7	04:08.2 ± 00:04.4	0:59:58.0 ± 00:13:43.1	1: 0.29
2	0:48:47.5 ± 10:39.9	04:20.2 ± 00:03.4	1:00:17.5 ± 00:13:46.6	1: 0.23
3	0:40:37.0 ± 05:42.2	04:36.7 ± 00:03.3	0:54:02.0 ± 00:39.2	1: 0.34
4	0:39:56.5 ± 02:46.6	04:08.9 ± 00:12.2	0:51:24.5 ± 01:43.9	1: 0.29
5	0:46:06.5 ± 10:49.8	04:05.5 ± 00:07.3	0:69:56.0 ± 13:45.9	1: 0.30
6*	0:44:23.0 ± 08:36.2	03:57.3 ± 00:02.9	0:59:33.5 ± 13:26.8	1: 034
Mean	0:44:24.1 ± 07:12.8	04:12.8 ± 00:14.0	0:57:31.9 ± 09:02.1	1: 0.30

Total repetitions duration, time (h) performing repetitions; Total session duration, Total Walking Time plus active recovery. Work: rest ratio, time (h) total walking time: active recovery during the key training sessions. *Athlete 6 performed an additional two key hyperhydration training sessions, additional data not presented.

#### Cooling Strategies

Internal and external cooling strategies were used prior to key training sessions and then replicated on the day of the men’s and women’s 20 km and men’s 50 km races during the Olympic Games. External pre-cooling comprised a mixed protocol of methods similar to those recently reported for race walking athletes prior to international championship events, and which have been reported to be effective for improving performance (Ross et al., 2013; Stevens et al., 2020; Racinais et al., 2021). Pre-cooling methods included a 30 min cold water immersion (CWI) protocol with a graded decrease in water temperature, and the application of ice-cold towels. Athletes selected the method to be used on the day of their race, based on their tolerance of the different pre-cooling methods when they were trialed during the team holding camp. Ice vests (Arctic Heat, Gold Coast, QLD, Australia) were then used by all athletes as they entered the call room prior to their Olympic Games events. Internal pre-cooling comprised ice slurries prepared by team sports dietitians, according to a ratio of 1 scoop sports drink (Powerade, Coca-Cola Company, Atlanta, GA, United States: 2 cups ice: 1 cup water, combined using a blender (Nutribullet, Los Angeles, CA, United States). Participants consumed up to 7 mL⋅kg^–1^ BM of ice slurry according to their personal preference. Ice slurry ingestion commenced 60 min prior to the training session or scheduled race start time and finished 30 min prior to the race start time as described previously (Ross et al., 2013). Internal and external strategies for use in-race were practiced during key training sessions, consistent with recently reported methods used during race walking events in major championship events (Scanlan et al., 2017; Stevens et al., 2020). External methods included cooling the skin with cold water and ice, placing ice within clothing, or held within the hands, and the application of cold towels, caps soaked in ice water and neck coolers made from ice vest gel inserts (Arctic Heat, Gold Coast, QLD, Australia). Meanwhile, in-race “sensory” cooling was facilitated via ingestion of menthol gels (0.7% concentration). The use of such gels was trialed during the team holding camp to allow personalized selection from a range of gels with varying menthol concentrations. Athletes selected from menthol gels after trialing different concentrations (0.1, 0.3, 0.5, and 0.7%). Internal and external cooling strategies were used at several time points in-race according to the individual’s chosen racing strategies.

#### Nutritional Interventions

Practice was undertaken prior to key training sessions with hyperhydration protocols involving glycerol and sodium chloride added to a fluid bolus. This involved both habituation to practices as well as identification of the ideal individualized protocol for use on the Olympic Games race day. Glycerol was ingested at a dose of 0.7–1.0 mL⋅kg^–1^; co-ingested with 17.85 mL⋅kg^–1^ water mixed with sports drink (Powerade, Coca-Cola Company, Atlanta, GA, United States or SiS GO Electrolyte, Science in Sport, London, United Kingdom) consistent with previously reported glycerol doses (van Rosendal et al., 2010) and current nutritional recommendations for athletics (Burke et al., 2019a), 180 min before the training session. Sodium chloride was ingested at a dose of 7.5 g⋅L^–1^, consistent with recently reported doses (Goulet et al., 2018). The dose was sub-divided into four equal volumes, ingested on a 20 min cycle, with each dose ingested within 5 min (Table 5). Hydration measures including plasma osmolality (mOsmol⋅kg^–1^); and body mass (kg) were recorded 180 min prior to ingestion, post-ingestion and immediately post-training. Gastrointestinal (GI) symptoms (Gaskell et al., 2019), were documented at the same time points as hydration measures, given the association between GI symptoms and hydration status (Rehrer et al., 1990).

**TABLE 5:** Hyperhydration protocol used prior to key training sessions (16th July and 23rd July 2021) during the team holding camp.

Athlete	Glycerol (mL⋅kg^–1^)	Sodium (g⋅L^–1^)	Water (mL⋅kg^–1^)	Sports drink intake (g)	Sports drink type*		Glycerol/Sodium bolus (mL)	Sports drink volume (mL)
					Session 1	Session 2		
1^†^	1.1 ± 0.1	7.5 ± 0.0	19.6 ± 2.5	28 ± 40	56 g Powerade	0 g	267.8 ± 24.3	1070.4 ± 95.7
2	0.0 ± 0.0	7.5 ± 0.0	16.4 ± 2.0	63 ± 10	70 g Powerade	56 g Powerade	226.6 ± 28.4	906.3 ± 113.3
3	1.1 ± 0.1	7.5 ± 0.0	19.6 ± 2.5	75 ± 7	70 g Powerade	80 g SiS GO	315.9 ± 39.7	1263.6 ± 158.7
4	1.1 ± 0.1	7.5 ± 0.0	19.6 ± 2.5	84 ± 0	84 g Powerade	84 g Powerade	344.2 ± 41.5	1376.7 ± 165.9
5	1.1 ± 0.1	7.5 ± 0.0	19.6 ± 2.5	84 ± 0	84 g Powerade	84 g Powerade	360.0 ± 43.4	1439.9 ± 173.6
6^‡^	0.0 ± 0.0	7.5 ± 0.0	23.2 ± 3.6	108 ± 16	84 g Powerade	80 g SiS GO and 36 g Hydralyte	410.8 ± 54.3	1643.0 ± 217.2
Mean	0.7 ± 0.5	7.5 ± 0.0	20.2 ± 3.2	79 ± 30			333.7 ± 75.0	1334.7 ± 299.9

Sports drink and glycerol/sodium were co-ingested over a 60-min period, with the dose sub-divided into four aliquots, ingested at 20 min intervals. *Sports drink listed as SiS GO Electrolyte (SiS Go) and Hydralyte Sport (Hydralyte). ^†^Consumed 131 mL of diet cordial with water in session 2. ^‡^Athlete 6 performed an additional two key training sessions. Sports drink intake for additional sessions were 80 g SiS GO and 36 g Hydralyte Sport for both sessions.

For specified athletes (*n* = 2), a 5-day low energy availability (EA) intervention was implemented and supervised by the team sports dietitians. The two athletes who used the LEA strategy had previously implemented similar strategies (managed by experienced sports dietitians) to achieve an ideal race body composition as part of their preparation for major international events. The daily EA target was set at 15–20 kg FFM^–1^⋅day^–1^ based upon the athlete’s training demands for each day, with additional nutrition strategies implemented, based on those recently established for endurance events in athletics (Burke et al., 2019a). Nutritional intake targets were expressed as energy intake in kcal⋅day^–1^ and kJ⋅day^–1^, and energy, protein and fat intake in g⋅day^–1^ and g⋅kg^–1^.day^–1^ (Table 6).

**TABLE 6:** Example of low energy availability (EA) intervention, with additional nutritional support for targeted sessions, during the team holding camp (*n* = 1; female).

Day	Training	Baseline EA target	Special nutrition strategies	Energy		Carbohydrate		Protein		Fat	
				Cal	kJ	g	g⋅kg^–1^	g	g⋅kg^–1^	g	g⋅kg^–1^
1	Gym	20		1,200	5,000	140	2.8	85	1.7	20	0.4
2	8 km walk	15	Carb refueling post-workout	1,946 200	8143	292 50	5.6	122	2.3	32	0.6
	Repetitions: 10 × 1,200 m		Rehearsal of pre-race glycerol hyperhydration	280		70					
3	12 km walk	15		1,369	5,727	205	3.9	86	1.6	23	0.4
4	25 km long walk	15	Carb refueling post-workout	2,466 400	10,318	370 100	7.0	154	2.9	41	0.8
	Repetitions: 10 × 400 m										
5	Gym	20		1,491	6,238	205	3.1	112	1.7	25	0.4
6	4 km walk	15		1,773	7,418	266	5.1	111	2.1	30	0.6
	Repetitions: 10 × 1 km		Carb refueling post-workout Rehearsal of pre-race glycerol hyperhydration	200 280		50 70					
**7**	16 km walk	15									
	8 km walk			2,053	8,826	309	5.9	129	2.5	34	0.7

*Daily energy intake expressed as calories (Cal) and kilojoules (kJ) per day; daily carbohydrate, protein and fat intake expressed as grams (g) and grams per kilogram (g⋅kg^–1^).*

#### Race Day Planning

Athletes were consulted throughout the development of different components of their race day plans (e.g., cooling strategies and nutritional interventions). As such, athletes were actively involved in the decision-making process that led to the development of their individual race day interventions and therefore did not provide data to describe their overall perceptions or feedback of their race day interventions upon completion of this observational study.

### Statistical Analyses

Data were reported using descriptive statistics (mean ± SD). Olympic Games race performances were reported, and the percentage change compared with the athlete’s personal best time, as documented within the World Athletics database (World Athletics, 2021), was calculated.

## Results

### Physiological and Training Data

The highest VO_2max_ result for female athletes was 63.6 mL⋅kg^–1^⋅min^–1^ and 73.7 mL⋅kg^–1^⋅min^–1^ for male athletes. The best outcome for race walking economy, defined as the lowest proportional oxygen use at 11 km⋅h^–1^ for females and 13 km⋅h^–1^ for males (Saunders and Green, 2012), was 61.2% VO_2max_ for female athletes and 63.4% VO_2max_ for male athletes. The highest velocity at VO_2max_ (vVO_2max_) was 17.5 km⋅h^–1^ for male athletes and 15.7 km⋅h^–1^ for female athletes The best 4 mmol⋅L^–1^ blood lactate walking speed was 80.2% vVO_2max_ for female athletes and 86.7% vVO_2max_ for male athletes. Training volume for the six athletes was the highest in the second week of the team holding camp, and training intensity was lowest in the first week of the team holding camp.

### Nutritional Interventions and Hydration

The highest post-exercise plasma osmolality observed was recorded for one athlete who did not complete the session (athlete 4), and the athlete with the lowest post-exercise body mass change (%) in comparison with pre-exercise data (athlete 3) completed a session that was lower in volume and intensity than the other athletes (Table 7). Minimal upper, middle, and lower gastrointestinal symptoms were recorded at pre, during and post-training time points (Table 8).

**TABLE 7:** Hydration measures from key training sessions (16th July and 23rd July 2021) during the team holding camp.

	Osmolality (mOsmol⋅kg^–1^)			Body mass (kg)				Fluid intake (mL)
Athlete	Pre-ingestion	Post-ingestion	Post-training	Pre-glycerol	Post-glycerol	Post-training	Change (%)	
1	291 ± 27	300 ± 13	311 ± 36	51.45 ± 0.21	52.55 ± 0.14	50.85*	−0.9*	450 ± 240
2^†^	286 ± 10	288 ± 13	306 ± 28	51.18 ± 0.25	51.95 ± 0.07	50.83 ± 0.18	−0.8 ± 0.1	385 ± 314
3	289 ± 6	299 ± 9	303 ± 9	56.90 ± 0.00	57.90 ± 0.21	57.10 ± 0.14	0.4 ± 0.2	762 ± 661
4	292 ± 9	303 ± 11	354 ± 84	62.38 ± 0.46	63.30 ± 0.57	61.90 ± 0.42	−0.8 ± 0.1	545 ± 218
5	277*	299 ± 11	305 ± 18	65.15 ± 0.07	66.34 ± 0.06	64.35 ± 0.21	−1.2 ± 0.4	506*
6^‡^	283 ± 10	296 ± 11	311 ± 24	65.05 ± 0.28	66.31 ± 0.02	64.70 ± 0.33	−1.0 ± 0.4	561 ± 527^§^
Mean ± SD	286 ± 11	297 ± 10	314 ± 34	59.59 ± 6.11	60.67 ± 6.23	59.85 ± 5.79	−0.7 ± 0.5	537 ± 329

Values provided indicate mean (±SD) values across the two key training sessions. *Data only available from one of the two key training sessions.

*^†^Sodium only hyperhydration protocol. ^‡^Athlete 6 performed an additional two training sessions. ^§^Data only available from two of the two key training sessions. Body mass change (%) indicates the percentage difference between pre-ingestion and post-training time points.*

**TABLE 8:** Gastrointestinal symptoms quantified during key training sessions during the team holding camp.

Training session	Pre-ingestion	Post-ingestion	Post-training
T1 UGI	2.00 ± 3.00	3.00 ± 3.75	1.00 ± 1.50
T2 UGI	0.00 ± 0.75	2.50 ± 1.75	0.00 ± 0.00
T1 LGI	1.5 ± 2.5	1.50 ± 4.00	1.00 ± 2.00
T2 LGI	0.00 ± 0.00	0.50 ± 1.75	1.00 ± 2.75
T1 OGI	0.00 ± 0.00	0.50 ± 1.00	0.00 ± 0.75
T2 OGI	0.00 ± 0.00	0.50 ± 1.00	0.00 ± 0.75
T1 Total	2.50 ± 4.00	3.50 ± 9.25	2.50 ± 3.25
T2 Total	0.00 ± 0.75	3.00 ± 5.50	2.00 ± 6.50

*Data presented as the Median ± IQR total score from the gastrointestinal (GI) symptoms questionnaire (Gaskell et al., 2019), comprised of upper (U), lower (L), and other (O) GI symptom questions from key training sessions 1 (T1) and 2 (T2).*

### Race Performance

Performance outcomes for the athletes within this study included 6th place in the women’s 20 km event (1:30:39), which was the athlete’s second-best performance time of all 20 km races within her career, and 8th in the men’s 50 km event (3:52:01), which was a personal best performance time. For the athlete who achieved 6th place in the 20 km event, her performance in the Olympic Games was a 2.0% performance decrement compared with her personal best performance (1:28:50). Additionally, one of the athletes achieved a personal best performance in the men’s 20 km event (1:24:00); Table 9.

**TABLE 9:** Olympic games performances and environmental conditions for Australian race walking athletes (*n* = 6).

Athlete	Event	Event date	Event start time	Temperature (°C) *Race start*	Temperature (°C) *Race finish*	Humidity (%) *Race start*	Humidity (%) *Race finish*	Performance (h)	Performance impairment (%)
1	20 km W	06/08/21	16:30	31	30	64	69	1:30:39	2.0
2	20 km W	06/08/21	16:30	31	30	64	69	1:38:11	8.9
3	20 km W	06/08/21	16:30	31	30	64	69	1:38:21	6.6
4	20 km M	05/08/21	16:30	31	30	63	73	1:27:55	4.6
5	20 km M	05/08/21	16:30	31	30	63	73	1:24:00	−0.5*
6	50 km M	06/08/21	05:30	25	30	86	79	3:52:01	−0.4*

Performance times presented for men’s (M) and women’s (W) 20 and 50 km events. Performance impairments calculated as the percentage difference from personal best times prior to the Olympic Games. *Athlete 5 achieved a personal best time during the Olympic Games men’s 20 km event, and athlete 6 achieved a personal best performance during the 50 km event.

## Discussion

This observational study provides novel data on the specific preparatory strategies of elite male and female endurance athletes competing in hot, humid conditions at the 2021 Tokyo Olympic Games. The athletes in this study utilized a combined approach of passive and active heat acclimation training prior to embarking on heat acclimatization training during the team holding camp, as well as intervention strategies targeting hydration, cooling, and body composition manipulation. This study combined the collection of both observational and self-report data and documents a careful integration of a broad range of strategies to support optimal performance in challenging environmental conditions.

The training volume completed within the team holding camp for the athletes within this observational study was similar to that previously reported for elite female and male race walkers (Pugliese et al., 2014; Espinel and Arjona, 2020) during their final preparations for Olympic Games. Further, the training volume and intensity were reduced throughout the heat acclimatization training completed during the team holding camp, as has been recommended to facilitate adaptations to heat and humidity whilst reducing physiological strain (Périard et al., 2015; Racinais et al., 2015). Specifically, weekly training volume was lower in the first week of the heat acclimatization training (∼90 km) compared with the following week of the holding camp (∼120 km). In addition, within high intensity sessions, training volume was limited to no more than 80% of the race distance prior to the team holding camp and was reduced from the first week of the holding camp (∼13 km) and then further reduced in the second week (12 km) and third week (10 km). Whilst a traditional tapering period was not possible due to the complex interactions between the requirements of heat acclimation and acclimatization training, physiological testing and travel to the team holding camp and Olympic Games, the athletes were able to complete a tapering period of ∼7 days at the end of the camp. The athletes within this observational study also completed one block of altitude training (21 days in December 2020) as an additional component of their preparation for the Olympic Games. This is different to the approach traditionally practiced by elite Australian endurance athletes in which comprehensive strategies involving natural altitude and “live high-train low” altitude-house protocols are implemented prior to major international events (Carr et al., 2020). However, COVID-19 travel restrictions on international travel for Australian athletes necessitated changes to usual practices. Furthermore, it allowed consideration of recent recommendations that sequential exposure to altitude and hot, humid conditions may provide greater performance benefits than concurrent exposure to both environmental stressors (Mujika et al., 2019). Although the current observational study provides an account of the integration of the training practices of elite athletes with heat acclimation and acclimatization training, further experimental research is required to quantify physiological responses to the sequential exposure to heat acclimation, acclimatization, and passive heat exposure within a periodized preparation for major international events, particularly when coupled with real-world performance outcomes.

The athletes in this observational study utilized a combined active and passive intermittent heat acclimation approach, over 6–7 weeks. As an example, one of the female athletes completed 13 passive and 14 active sessions over 7 weeks. Although this strategy was not strictly consistent with recent recommendations suggesting a minimum 6–7 consecutive days of heat exposure training, or an optimal acclimatization period of ∼14 days (Racinais et al., 2015), the athletes attained an additional 18 days of continuous passive and active exposure to hot and humid conditions during the team holding camp. The athletes therefore completed 23 sport-specific, heat exposure training sessions over 18 days, and although athletes trained in the morning and afternoon during the training camps to allow sufficient rest between sessions, they were still exposed to temperatures of ∼25°C and 73% RH for several hours. The athletes tolerated these training sessions well, as suggested by their maintenance of required pace during repeat efforts. Furthermore, athletes were passively exposed to the heat and humidity in day-to-day activities throughout the holding camp. The number of days and duration of exposure, as well as the temperature and humidity, are therefore within that previously reported to be beneficial in elite athletes (Périard et al., 2017). Such active and passive heat acclimation is expected to induce changes such as reductions to exercising heart rate, improvements in thermal comfort and perception of effort, and changes to sweat rates (Périard et al., 2015). The athletes also undertook pre-cooling prior to their key hyperhydration training sessions to further prepare them for their race day protocols. While pre-cooling has been shown as advantageous to performance in the heat (Ross et al., 2013), it also does not appear to attenuate adaption to HA training (Choo et al., 2020). While it was beyond the scope of this study to determine if these adaptations took place, given the volume of observational data collected during the team holding camp, this consideration provides a basis for important future research in elite and well-trained endurance athletes when preparing for races in hot, humid conditions.

Hydration measures collected within the current study indicated that the increase in plasma osmolality was similar in magnitude to that previously reported after ingestion of hyperhydration agents (Riedesel et al., 1987; Sims et al., 2007a), and the change in participants’ body mass indicated adequate hydration (McDermott et al., 2017; Périard et al., 2021). Whilst hydration guidelines have changed across recent decades, the avoidance of excessive dehydration (e.g., >2% change in body mass) is still considered a desirable target if practical, due to the associations between dehydration and reduced plasma volume, cardiac drift, increased thermal stress, gastrointestinal distress and impaired exercise performance (Rehrer et al., 1990; Montain and Coyle, 1992; Cheuvront et al., 2005; McDermott et al., 2017). The increase in plasma osmolality reported in the current study was similar to that reported for both glycerol and sodium ingestion when ingested in isolation (Riedesel et al., 1987; Sims et al., 2007a), although the elevation was sustained for a longer time period than that previously reported with glycerol ingestion (Riedesel et al., 1987). Further, the glycerol and sodium ingestion protocols were well tolerated by all athletes, with minimal GI symptoms reported immediately following ingestion and following their training sessions. Although it is beyond the scope of this observational study to make mechanistic speculations, results of the current investigation could be associated with factors such as the shorter ingestion protocol (1 h) used in the current investigation compared with previous reports after glycerol ingestion (Riedesel et al., 1987), and the different physiological mechanisms that elicit glycerol and sodium-induced hyperhydration (Robergs and Griffin, 1998). There is scope for future research investigating additional hydration measures (e.g., urine specific gravity, urine color) after combined sodium and glycerol ingestion, coupled with effects on elite athletes’ real-world performance. Such investigations would add to the existing evidence of improvements in laboratory-based exercise capacity in well-trained participants after ingestion of osmotic agents (Anderson et al., 2001; Sims et al., 2007a).

The intervention involving a brief period of exposure to LEA for several athletes within the current study was implemented as a periodized strategy for body composition management under the guidance and supervision of experienced sports dietitians, and with the athletes’ consent. A reduction in body mass/body fat can improve relative VO_2max_, exercise economy, and cooling capacity, all of which may be associated with enhanced performance in endurance events (Dennis and Noakes, 1999; Stellingwerff, 2018). Importantly, structured and supervised short-term low energy availability interventions also reduce the serious health and performance risks associated with sustained or inadvertent low energy availability, including reduced insulin, reduced pulse frequency of luteinizing hormone and increased cortisol, which are associated with menstrual dysfunction, increased injury frequency and compromised bone health (Tenforde et al., 2017; Areta et al., 2021). The integration of this intervention with the periodized approach to training and heat exposure, as well as cooling and hydration interventions was part of a comprehensive preparation strategy which was designed to improve athletes’ capacity to avoid the health risks and performance impairments associated with substantial dehydration and increased core temperature when racing in hot, humid conditions (Burke et al., 2019b). Further, athletes were actively involved in the trials and decision-making that led to the development of their individual race-day strategies used during the Olympic Games. The two athletes who completed the low energy availability intervention as part of their preparation achieved Olympic Games performances that were either personal best performances or within 2% of their personal best performance times despite the environmental conditions. These results compared favorably with performance decrements of more than 5% for top 10 competitors across all race walking and marathon events in the Tokyo Olympic Games compared with personal best times (World Athletics, 2021), and reported decrements in marathon runners’ performance (3%) in past World Athletics Championship events held in hot conditions (Guy et al., 2015).

This study is one of the first to comprehensively detail the preparatory strategies of elite endurance athletes for competition in hot and humid conditions at an Olympic Games. Nevertheless, several limitations are noted including the small sample size. However, two of the six finished within the top eight within their respective events, and therefore this study may provide some insight into the individual preparation strategies of high-performance athletes. Furthermore, the quantification of internal measures such as heart rate was not included within the current investigation, as the athletes within the observational study do not typically wear heart rate monitors during training or racing. Rather, training for this group of athletes is typically prescribed using external measures including training volume (km) and speed (km⋅h^–1^ and min). Additionally, we unfortunately obtained erroneous core temperature data from core pills, thereby limiting any inferences in the athlete’s heat acclimatization status. These data could have provided further insight into the ability of elite endurance athletes to undergo adaptations to heat exposure training prior to an international event. However, the findings of the current study provide some important practical recommendations for elite endurance athletes preparing for events in hot and humid conditions. Systematically planned and periodized heat acclimation over a period of 6–7 weeks, using a combination of passive and active sessions in an intermittent fashion may allow athletes to prepare for more challenging heat acclimatization training sessions leading into holding camps or pre-event training blocks that utilize the continuous HA approach. The combination of hyperhydration with internal and external cooling prior to key training sessions in the heat might allow athletes to simulate expected race day conditions and pacing strategies. It should be noted, however, that the athletes in this study refined their heat acclimation and acclimatization, cooling and hyperhydration strategies, under the management of coaches, sport scientists and dietitians, over a period of ∼2–3 years, facilitating the development of a combination of heat mitigation strategies that were tailored to athletes’ specific needs (e.g., training history, capacity to ingest different supplements). This individualized approach culminated in well-established routines that were implemented for each athlete during the holding camp and Olympic Games.

## Conclusion

We recommend that elite race walkers preparing to compete in hot, humid conditions perform a combination of heat exposure training, including intermittent heat acclimation (1–2 sessions per week) and passive heat exposure (1–2 sessions per week) 6–7 weeks prior to a heat acclimatization training period, ideally conducted concurrently with a team holding camp utilizing continuous HA training. On the day of key training sessions and major races, cooling strategies and hydration/hyperhydration strategies should be implemented prior to and during races, as additional heat mitigation strategies. A periodized nutrition strategy can be implemented, under the guidance of experienced sports dietitians, to modify body composition, as part of a comprehensive, periodized preparation for championship races held in hot, humid conditions.

## Data Availability Statement

The raw data supporting the conclusions of this article will be made available by the authors, without undue reservation.

## Ethics Statement

The studies involving human participants were reviewed and approved by the Central Queensland University Ethics Committee. Written informed consent for participation was not required for this study in accordance with the national legislation and the institutional requirements. Written informed consent was not obtained from the individual(s) for the publication of any potentially identifiable images or data included in this article.

## Author Contributions

AC, BV, JR, AR, LB, and JG contributed to the study design, data collection, data analysis, and manuscript preparation and revision. All authors agreed to be accountable for the content of the work.

## Conflict of Interest

The authors declare that the research was conducted in the absence of any commercial or financial relationships that could be construed as a potential conflict of interest.

## Publisher’s Note

All claims expressed in this article are solely those of the authors and do not necessarily represent those of their affiliated organizations, or those of the publisher, the editors and the reviewers. Any product that may be evaluated in this article, or claim that may be made by its manufacturer, is not guaranteed or endorsed by the publisher.
