# Supplementary material for: Competing in Hot Conditions at the Tokyo Olympic Games: Preparation Strategies Used by Australian Race Walkers
Source: Front Physiol. 2022 Mar 23;13:836858. doi: 10.3389/fphys.2022.836858 (PMC8983867; doi:10.3389/fphys.2022.836858)
Supplement: Supplementary file 1 [file Table_1.pdf]

SUPPLEMENTARY TABLE 1 | Example of training performed during the team holding camp (*n* = 1; female).

	Monday	Tuesday	Wednesday	Thursday	Friday	Saturday	Sunday
**WEEK -4 Pre-Olympic Games; Volume = 110km; 15.8% > 4mmol·L^{-1}**							
**AM**	Travel to Cairns	10km Walk *5:11min·km^{-1}*	Lab Testing	Easy 6km Run + Gym *4:47min·km^{-1}*	Easy 12km Walk *5:07min·km^{-1}*	Easy 12km Walk + 30min Sauna *5:08min·km^{-1}*	20km Walk *4:58min·km^{-1}*
**PM**		Easy Run *4:52min·km^{-1}*	10km Walk *5:14min·km^{-1}*		Repetitions: *8x1609m**		Repetitions: *10x300m*
**WEEK -3 Pre-Olympic Games; Volume = 125km; 17.9% > 4mmol·L^{-1}**							
**AM**	Easy Run + Gym	Easy 12km Walk + 30min Sauna *5:07min·km^{-1}*	22km Walk *4:52min·km^{-1}*	Gym	Easy 8km Walk *5:09min·km^{-1}*	Easy Walk + Sauna	24km Walk *4:56min·km^{-1}*
**PM**		2x8x400m *3:45min·km^{-1}*	Easy Run + Sauna		Repetitions: *10x1200m**		Repetitions: *10x400m*
**WEEK -2 Pre-Olympic Games; Volume = 72km; 16.7% > 4mmol·L^{-1}**							
**AM**	6km Run + Gym	Warm-up	Easy 12-20km Walk	Gym	Warm-up	Travel to Sapporo	Warm-up
**PM**		Repetitions: *10x1km (4:10)*	8km (Walk/Run)		Travel to Sydney	Light Training	Easy 6-8km Walk

Hyperhydration trial.
