# Supplementary material for: Competing in Hot Conditions at the Tokyo Olympic Games: Preparation Strategies Used by Australian Race Walkers
Source: Front Physiol. 2022 Mar 23;13:836858. doi: 10.3389/fphys.2022.836858 (PMC8983867; doi:10.3389/fphys.2022.836858)
Supplement: Supplementary file 2 [file Table_2.pdf]

SUPPLEMENTARY TABLE 2 | Example of training performed during the team holding camp (*n* = 1; male).

	Monday	Tuesday	Wednesday	Thursday	Friday	Saturday	Sunday
**WEEK -3 Pre-Olympic Games; Volume = 110km; 16.2% > 4mmol·L^{-1}**							
**AM**	Travel to Cairns	Easy 10km Walk	Lab Testing	Easy 8km Walk *5:01min·km^{-1}*	Easy 12km Walk *5:09min·km^{-1}*	Easy 12km Walk *5:10min·km^{-1}*	33km Walk *4:56min·km^{-1}*
**PM**	Gym		Easy 8km Walk *5:01min·km^{-1}*		Repetitions*† *8x1609m*		Repetitions: *10x300m*
**WEEK -2 Pre-Olympic Games; Volume = 120km; 18.7% > 4mmol·L^{-1}**							
**AM**	Easy 6km Run + Gym	15km Walk*† *5:04min·km^{-1}*	22km Walk *4:50min·km^{-1}*	Gym	Easy 8km Walk *5:08min·km^{-1}*	Easy 12km Walk *5:16min·km^{-1}*	25km Walk *4:48min·km^{-1}*
**PM**		Repetitions: *2x8x400m*	Easy 9km Run *4:56min·km^{-1}*		Repetitions*† *10x1200m*		Repetitions: *10x400m*
**WEEK -1 Pre-Olympic Games; Volume = 72km; 23.6% > 4mmol·L^{-1}**							
**AM**	Rest Day	15km Walk *4:27min·km^{-1}*	18km Walk *4:58min·km^{-1}*	6km Run + Gym *4:16min·km^{-1}*	Warm-up	Travel to Sapporo	Warm-up
**PM**		5km Run *4:25min·km^{-1}*	Easy 7km Run *4:16min·km^{-1}*		Travel to Sydney	Easy 6km Walk *5:01min·km^{-1}*	Easy 5km Walk *5:07min·km^{-1}*

**Hyperhydration trial. †Pre-cooling trial*
